# Revisiting the link between NADPH oxidase p22phox C242T polymorphism and ischemic stroke risk: an updated meta-analysis

**DOI:** 10.1515/biol-2025-1221

**Published:** 2025-12-30

**Authors:** Yan Wang, Li An

**Affiliations:** Department of Blood Transfusion, Nanjing BenQ Medical Center, The Affiliated BenQ Hospital of Nanjing Medical University, Nanjing, Jiangsu, People’s Republic of China; Department of Gerontology, Zhongda Hospital Southeast University, Nanjing, People’s Republic of China

**Keywords:** ischemic stroke, NADPH oxidase, polymorphism, p22phox

## Abstract

The relationship between the NADPH oxidase p22phox C242T and susceptibility to ischemic stroke (IS) has been extensively studied, yet the findings from these studies remain inconsistent. To clarify this association, we conducted an updated meta-analysis of case-control studies. A comprehensive literature search of PubMed, Embase, and CNKI was performed on January 31, 2025. Studies exhibiting Hardy-Weinberg equilibrium (HWE) deviations in the control population were excluded from analysis. Pooled odds ratios (ORs) with corresponding 95 % confidence intervals (CIs) were calculated to evaluate the association across various genetic models. Our meta-analysis included 10 studies, comprising 3,422 cases and 3,410 controls. We found no significant association between the C242T polymorphism and IS susceptibility under any genetic model. Subgroup analyses stratified by ethnicity and IS type similarly revealed no significant increase in risk. Sensitivity analysis confirmed the stability of these pooled results. This updated meta-analysis suggests that the NADPH oxidase p22phox C242T polymorphism lacks a significant association with ischemic stroke risk. Nevertheless, larger and more diverse population studies are warranted to confirm these findings.

## 1 Introduction

Ischemic stroke (IS) is a complex multifactorial disorder resulting from the interplay of genetic predispositions and environmental factors such as hypertension, diabetes, hyperlipidemia, and smoking [1]. As a major global public health concern, IS imposes a substantial burden on families, communities, and healthcare systems. Genetic factors are increasingly recognized as key contributors to IS susceptibility, with several gene polymorphisms implicated in its pathogenesis [2], [3], [4]. Atherosclerosis, a primary cause of ischemic cerebrovascular disease, involves accelerated lipid peroxidation driven by elevated levels of reactive oxygen species (ROS) [5], [6], [7], [8]. A major enzymatic source of ROS is the nicotinamide adenine dinucleotide phosphate (NADPH) oxidase complex [9].

The NADPH oxidase complex, which includes membrane subunits such as gp91phox (NOX2) and p22phox, as well as cytosolic components like p47phox, p67phox, p40phox, and Rac2, is a key source of ROS production [10], [11], [12]. The *CYBA* gene, which encodes the p22phox protein, is located on the long arm of chromosome 16 and exhibits several allelic variants [13], [14], [15]. One notable variant is the C242T polymorphism, which involves an amino acid substitution (His/Tyr) at residue 72, causing significant morphological changes in the extracellular loop of the p22phox protein. This polymorphism may disrupt the interaction between p22phox and the catalytic subunit *NOX2*, potentially reducing ROS production by NADPH oxidase [16].

In the past few decades, numerous studies have investigated the relationship between the p22phox C242T polymorphism and the risk of ischemic cerebrovascular disease [17], [18], [19]. However, results have been inconsistent, with some studies suggesting an association and others reporting none [20], [21], [22]. This inconsistency is further complicated by potential population stratification and deviations from Hardy-Weinberg equilibrium (HWE) in control groups. Therefore, the objective of this updated meta-analysis was to quantitatively synthesize the most recent evidence to reassess the association between the NADPH oxidase p22phox C242T polymorphism and ischemic stroke risk.

## 2 Materials and methods

### 2.1 Literature search

We conducted an extensive electronic search of relevant studies in PubMed, Embase, and the China National Knowledge Infrastructure (CNKI) up to January 2025. Search keywords included “NADPH,” “p22phox,” “C242T,” “rs4673,” “polymorphism,” and “ischemic stroke.” Our aim was to identify all eligible case-control studies exploring the association between the p22phox C242T polymorphism and ischemic stroke (IS) susceptibility.

### 2.2 Selection criteria and quality assessment

Studies were included if they met the following criteria: (1) case-control design evaluating the association between the p22phox C242T polymorphism and IS susceptibility; and (2) provision of sufficient data on genotype and allele frequencies. Studies were excluded if: (1) control groups deviated from Hardy-Weinberg equilibrium (HWE); or (2) they were duplicate publications. Maintaining HWE in control groups is essential for minimizing genotype errors and preventing population stratification biases. Two authors independently assessed the quality of included articles using the Newcastle-Ottawa Scale, which encompasses eight key criteria: adequate case determination, representativeness of cases, control selection, control determination, comparability between cases and controls, exposure ascertainment, uniform ascertainment methods for both groups, and non-response rate [23].

### 2.3 Data extraction

For each study, we extracted the following information: first author’s surname, publication year, ethnic group, country of origin, sample size, IS subtype, and genotype frequency. This detailed data extraction enabled us to compile comprehensive information for our meta-analysis.

### 2.4 Statistical analysis

Given that some studies reported zero frequency for the TT genotype, we applied four genetic models for analysis: the allelic model (T allele vs. C allele), the dominant model (TT + TC vs. CC), the overdominant model (TC vs. TT + CC), and the codominant model (TC vs. CC). We calculated odds ratios (ORs) and 95 % confidence intervals (CIs) from each study to assess the association between the p22phox C242T polymorphism and IS susceptibility. Heterogeneity across studies was evaluated using the chi-square test and I^2^ statistic. Meta-regression was performed using the method of moments to identify sources of heterogeneity. Both fixed-effects and random-effects models were applied and compared to ensure robustness. Subgroup analyses were conducted based on IS subtypes and ethnicity to explore potential differences. Sensitivity analysis was performed by sequentially removing individual studies to assess the stability of the pooled results. Publication bias was evaluated using funnel plots and Egger’s regression test. Statistical analyses were conducted using MetaGenyo, a web-based tool for meta-analysis of genetic association studies (https://metagenyo.genyo.es/). If publication bias was detected, the trim-and-fill method was applied using the R package ‘meta’ to assess whether this bias affected the combined effect size results.

## 3 Results

### 3.1 Literature search and study characteristics

**Figure 1:**
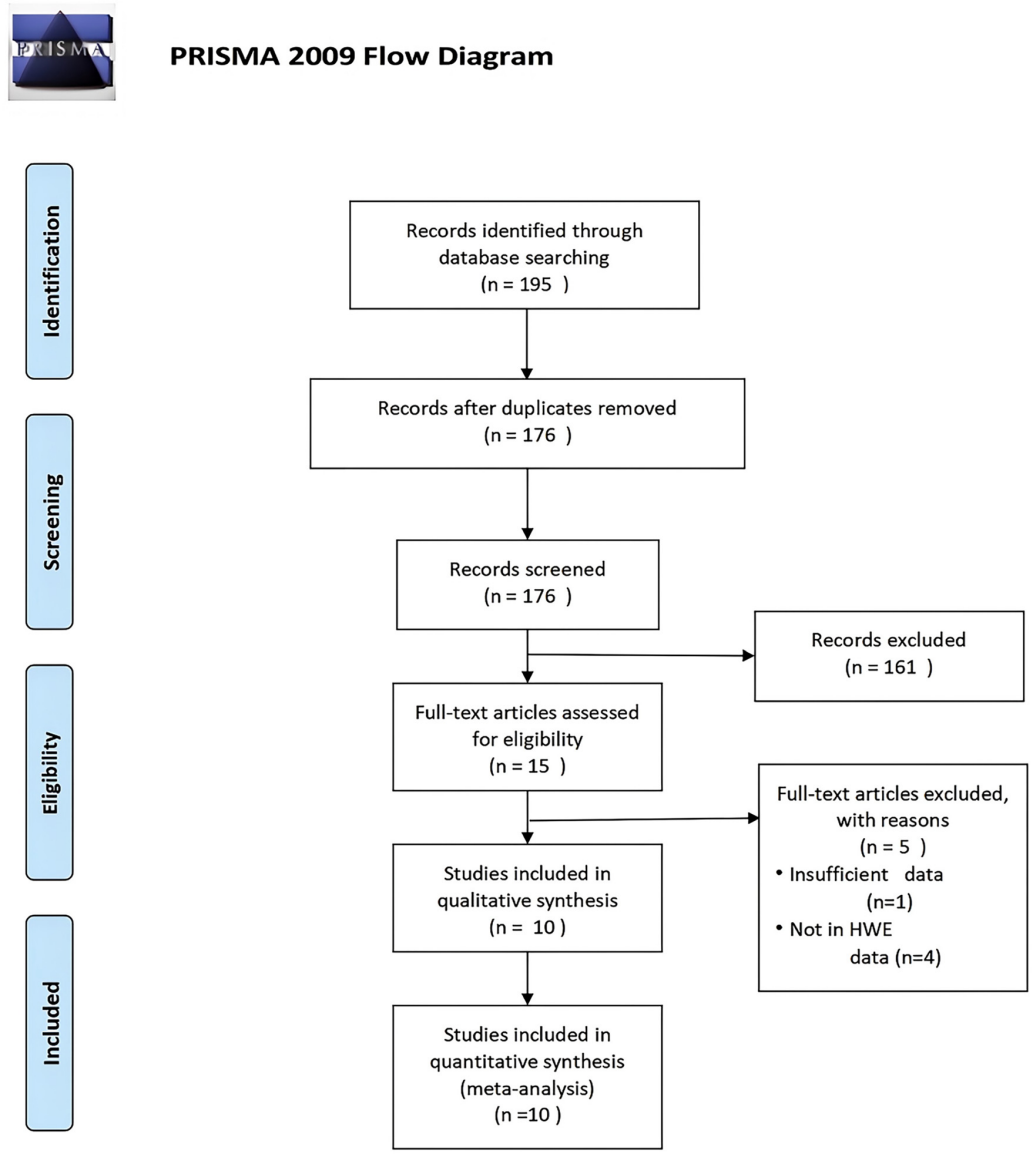
Flow of studies through the review process.

Our comprehensive search across PubMed, Embase, and CNKI identified 195 articles. After screening titles and abstracts, we selected 15 studies for detailed evaluation [17], [18], [19, [24], [25], [26], [27], [28], [29], [30], [31], [32], [33], [34], [35]. Upon thorough review, five articles were excluded based on predefined criteria [17], [18], [19, 24], 32] (Supplementary data). Specifically, one study lacked sufficient genotype and allele frequency data [17], while four others deviated from HWE in their control groups [18], 19], 24], 32]. Ultimately, 10 studies were included in our meta-analysis, encompassing 3,422 cases and 3,410 controls [25], [26], [27], [28], [29], [30], [31, [33], [34], [35] (Figure 1). The characteristics of these studies are summarized in Table 1, and the detailed distribution of alleles and genotypes is presented in Table 2. Following rigorous quality assessment, all included studies were rated as medium or high quality, with scores ranging from 5 to 9.

**Table 1:** Baseline characteristics of included studies.

First author, Year of publication	Country	Ethnicity	Sample size		Age (Mean ± SD, y)		Subtype of IS
			Case	Control	Case	Control	
Han [25]	China	East Asian	76	105	64.92 ± 11.1	63.91 ± 11.9	SVO and LAA
Ito [26]	Japan	East Asian	203	301	58 ± 8	59 ± 4	SVO and LAA
Jiang [35]	China	East Asian	185	183	66.31 ± 10.82	67.91 ± 12.27	SVO and LAA
Kovaleva [33]	Russia	Caucasian	746	500	67.89 ± 0.42	54.42 ± 0.55	SVO and LAA
Kuroda [27]	Japan	East Asian	1,055	1,055	70 ± 10	70 ± 10	SVO, LAA, CE and SUE
Li [21], 28]	China	East Asian	125	147	65.42 ± 11.65	65.22 ± 9.69	SVO and LAA
Li [34]	China	East Asian	284	335	61.46 ± 10.52	60.17 ± 9.56	SVO and LAA
Niemierc [29]	Poland	Caucasian	70	50	8.48 ± 5.44	9.0 ± 6.1	SVO, LAA and SUE
Shimo-Nakanishi [30]	Japan	East Asian	120	177	61.2 ± 11.4	58.9 ± 9.25	SVO and LAA
Yan [31]	China	East Asian	558	557	61.0 ± 9.8	62.2 ± 9.3	SVO and LAA

IS, ischemic stroke; SVO, small-vessel occlusion; LAA, large-artery atherosclerosis; CE, cardioembolism; SUE, stroke of undetermined etiology.

**Table 2:** Distribution of p22phox C242T genotypes and allelic frequency.

Study First author (years)	Distribution of p22phox C242T genotype						Frequency of p22phox C242T alleles				HWE P Value
	CC		CT		TT		C		T		
	Cases *n*	Controls *n*	Cases *n*	Controls *n*	Cases *n*	Controls *n*	Cases *n*	Controls *n*	Cases *n*	Controls *n*	
Han [25]	61	99	15	6	0	0	137	204	15	6	0.763
Ito [26]	158	261	42	38	3	2	358	560	48	42	0.634
Jiang [35]	162	173	20	10	3	0	344	356	26	10	0.704
Kovaleva [33]	351	240	302	206	93	54	1,004	686	488	314	0.329
Kuroda [27]	851	840	189	198	15	17	1,891	1,878	219	232	0.181
Li [21], 28]	113	144	11	3	1	0	237	291	13	3	0.900
Li [34]	231	261	51	71	2	3	513	593	55	77	0.444
Niemierc [29]	35	26	27	18	8	6	97	70	43	30	0.312
Shimo-Nakanishi [30]	102	154	18	23	0	0	222	331	18	23	0.355
Yan [31]	487	471	71	85	0	1	1,045	1,027	71	87	0.158

### 3.2 Meta-analysis findings

**Figure 2:**
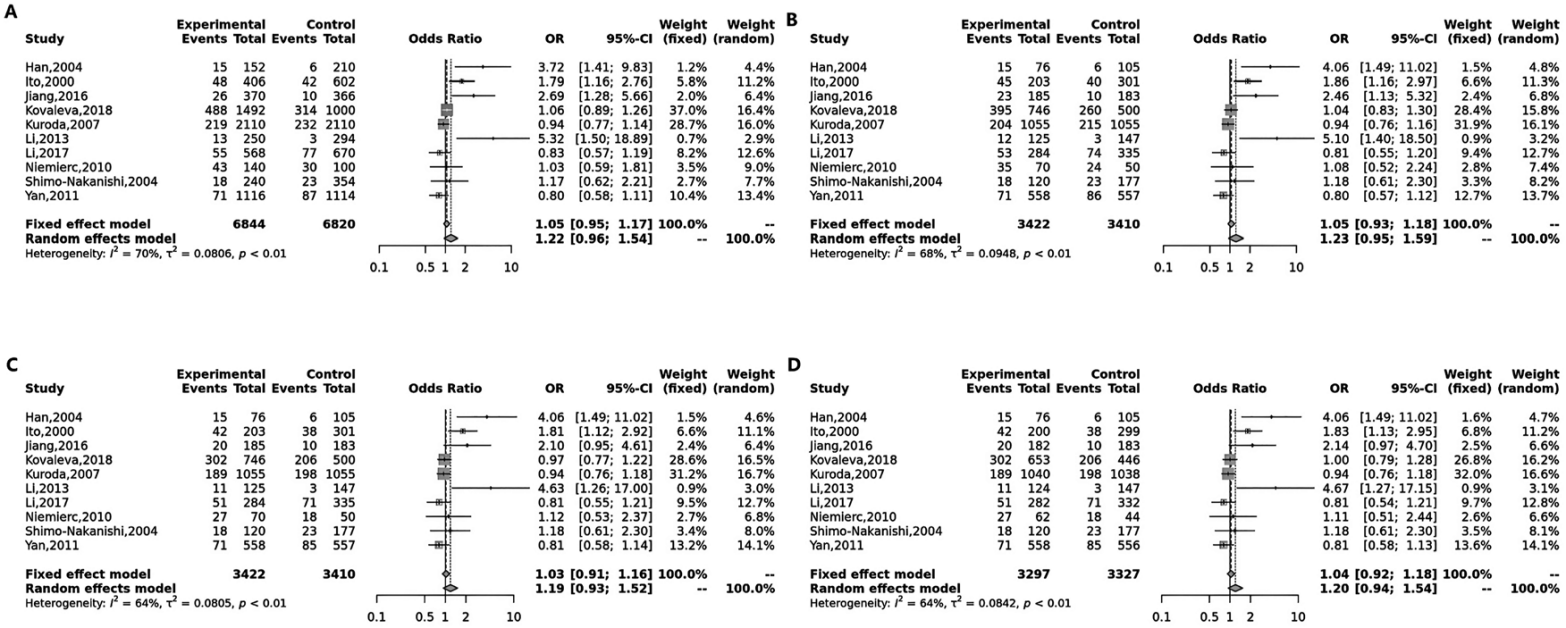
Forest plot analysis evaluating the association between NADPH oxidase p22phox C242T polymorphism and ischemic stroke (IS) risk. The analysis includes four genetic models: (A) Allelic model comparing the T allele to the C allele; (B) dominant model combining TT and TC genotypes against CC genotype; (C) overdominant model comparing TC genotype to the combined TT and CC genotypes; (D) codominant model comparing TC genotype to CC genotype.

Our meta-analysis revealed no significant association between the p22phox C242T polymorphism and ischemic stroke susceptibility in any genetic model. Specifically, the allelic model (T vs. C) showed an odds ratio (OR) of 1.22 with a 95 % confidence interval (CI) of 0.96–1.54 (p = 0.11). The dominant model (TT + TC vs. CC) yielded an OR of 1.23 (95 % CI: 0.95–1.59, p = 0.11), while the overdominant model (TC vs. TT + CC) had an OR of 1.19 (95 % CI: 0.93–1.52, p = 0.17). The codominant model (TC vs. CC) resulted in an OR of 1.20 (95 % CI: 0.94–1.54, p = 0.15). These results are visually represented in Figure 2.

### 3.3 Heterogeneity and subgroup analyses

Significant heterogeneity was observed across all genetic models in the overall analysis, with I^2^ statistics exceeding 50 % and p-values below 0.05. Subsequent meta-regression analysis failed to identify any significant influence of ethnicity or IS type on IS risk. Subgroup analyses stratified by ethnicity and IS subtype similarly revealed no significant associations under any genetic model (Table 3). Notably, heterogeneity markedly decreased in studies focusing on small-vessel occlusion and large-artery atherosclerosis (LAA) and no heterogeneity was observed in Caucasians (Supplementary data), suggesting that these factors may have distinct genetic influences.

**Table 3:** Odds ratios (ORs) and heterogeneity results for the genetic contrasts of C242T for IS.

	Population	OR				I^2^ (%)	*P-val* Q test	*P-val* Egger’s test
		Fixed effects (95 % CI)	*P*	Random effects (95 % CI)	*P*			
Allele contrast (T vs. C)	All	1.05 [0.95–1.17]	0.35	1.22 [0.96–1.54]	0.10	70.43	0.0004	0.0352
	Caucasian	1.06 [0.90–1.25]	0.49	1.06 [0.90–1.25]	0.49	0	0.9298	NA
	East Asian	1.05 [0.91–1.20]	0.51	1.37 [0.97–1.93]	0.07	76.98	0.0001	0.0173
	Japanese	1.05 [0.89–1.25]	0.55	1.22 [0.78–1.89]	0.38	72.03	0.028	0.4829
	Chinese	1.03 [0.83–1.29]	0.76	1.70 [0.90–3.20]	0.10	82.79	0.0001	0.0023
	Adult	1.05 [0.95–1.17]	0.35	1.25 [0.97–1.61]	0.09	73.71	0.0002	0.0347
	LAA	1.03 [0.85–1.25]	0.75	1.07 [0.80–1.44]	0.15	43.83	0.1485	0.5224
	SVO	1.08 [0.88–1.31]	0.47	1.11 [0.75–1.63]	0.61	61.38	0.051	0.8318
Dominant model (TT + TC vs. CC)	All	1.05 [0.93–1.18]	0.45	1.23 [0.95–1.59]	0.11	68.47	0.0008	0.0188
	Caucasian	1.05 [0.84–1.29]	0.71	1.05 [0.84–1.29]	0.71	0	0.9139	NA
	East Asian	1.05 [0.90–1.22]	0.51	1.22 [0.96–1.54]	0.10	75.47	0.0002	0.0165
	Japanese	1.06 [0.88–1.28]	0.52	1.23 [0.78–1.96]	0.37	70.93	0.0321	0.4755
	Chinese	1.03 [0.82–1.30]	0.79	1.67 [0.88–3.17]	0.12	81.49	0.0002	0.0022
	Adult	1.05 [0.93–1.18]	0.46	1.26 [0.95–1.65]	0.10	71.97	0.0004	0.0169
	LAA	1.03 [0.83–1.27]	0.82	1.10 [0.77–1.56]	0.61	53.83	0.0897	0.4429
	SVO	1.09 [0.88–1.35]	0.43	1.11 [0.75–1.65]	0.60	57.36	0.0708	0.8637
Overdominant model (TC vs. TT + CC)	All	1.03 [0.91–1.16]	0.67	1.19 [0.93–1.52]	0.16	64.06	0.0029	0.0121
	Caucasian	0.98 [0.79–1.22]	0.87	0.98 [0.79–1.22]	0.87	0	0.7277	NA
	East Asian	1.05 [0.90–1.22]	0.53	1.32 [0.94–1.84]	0.11	71.65	0.0009	0.0163
	Japanese	1.07 [0.88–1.29]	0.51	1.22 [0.79–1.88]	0.37	65.88	0.0533	0.467
	Chinese	1.02 [0.81–1.30]	0.86	1.57 [0.86–2.88]	0.14	78.67	0.0009	0.0026
	Adult	1.03 [0.90–1.16]	0.70	1.21 [0.93–1.57]	0.16	67.99	0.0016	0.0115
	LAA	1.02 [0.82–1.26]	0.88	1.11 [0.76–1.62]	0.60	59.22	0.0614	0.3985
	SVO	1.10 [0.88–1.37]	0.41	1.10 [0.77–1.58]	0.59	48.46	0.1207	0.9248
Codominant model (TC vs. CC)	All	1.04 [0.92–1.18]	0.56	1.20 [0.94–1.54]	0.15	68.47	0.0004	0.014
	Caucasian	1.01 [0.80–1.27]	0.92	1.01 [0.80–1.27]	0.92	0	0.8002	NA
	East Asian	1.05 [0.90–1.22]	0.53	1.32 [0.94–1.86]	0.10	72.23	0.0007	0.0162
	Japanese	1.07 [0.88–1.29]	0.51	1.22 [0.79–1.90]	0.37	67.18	0.0475	0.4683
	Chinese	1.02 [0.81–1.29]	0.86	1.58 [0.86–2.91]	0.14	78.98	0.0008	0.0026
	Adult	1.04 [0.91–1.18]	0.59	1.22 [0.96–1.54]	0.14	70.43	0.0004	0.0352
	LAA	1.02 [0.82–1.26]	0.86	1.11 [0.76–1.61]	0.60	58.41	0.0654	0.4036
	SVO	1.10 [0.88–1.37]	0.41	1.11 [0.77–1.60]	0.59	50.22	0.1103	0.908

IS, ischemic stroke; SVO, small-vessel occlusion; LAA, large-artery atherosclerosis; Not applicable, NA.

### 3.4 Sensitivity and publication bias analyses

**Figure 3:**
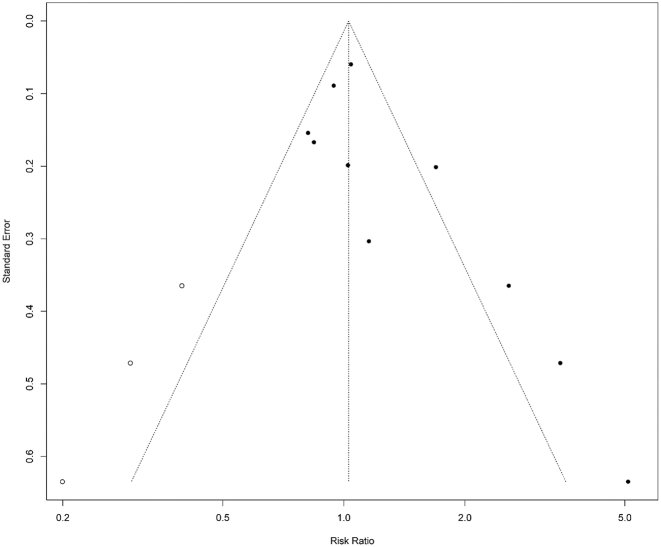
The adjusted funnel plot for the allelic model following adjustment with the trim-and-fill method. Solid circles denote the original studies, while hollow circles represent the imputed ones, respectively.

Sensitivity analysis, performed by sequentially removing individual studies, indicated that our findings were robust, with no single study significantly influencing the overall results. Publication bias was assessed using funnel plots and Egger’s regression test. While publication bias was detected in all genetic models (p-values ranging from 0.012 to 0.035), it was absent in studies of small-vessel occlusion and large-artery atherosclerosis. The results remained unchanged after adjusting for publication bias using the trim-and-fill method in the allelic model (OR 1.027, 95 % CI 0.693–1.523; p = 0.892; Figure 3).

## 4 Discussion

Ischemic stroke (IS) is a complex pathological condition characterized by reduced blood flow and oxygen supply to brain tissue. The precise mechanisms linking NADPH oxidase to ischemic stroke remain unclear, although reactive oxygen species (ROS) have been implicated in its pathophysiology [10], 36]. NADPH oxidase is a key enzyme complex responsible for ROS production in the brain [36], 37]. Previous studies have suggested that superoxide-producing NADPH oxidases are associated with ischemia-reperfusion injury in the brain [38]. The C242T polymorphism in the p22phox subunit may disrupt the interaction between p22phox and *NOX2* [16], potentially reducing ROS production [39], 40]. However, our meta-analysis did not identify a significant association between the p22phox C242T polymorphism and the risk of ischemic stroke, despite the presence of publication bias. This bias typically stems from the systematic under-reporting of small-scale studies with null or negative findings, consequently leading to an overestimation of the true effect size within meta-analyses. Therefore, the pooled ORs observed before adjustment should be interpreted as representing the probable upper bound of any potential association. Crucially, however, the robustness of our primary finding, a demonstrable lack of significant association, is robustly reinforced by both sensitivity analyses and the trim-and-fill method. The application of the trim-and-fill method, which imputes theoretically missing studies to correct funnel plot asymmetry, resulted in an adjusted effect size even closer to the null value (OR 1.027) that remained non-significant. This compellingly suggests that, even after accounting for potential publication bias, the conclusion of no association remains statistically robust.

Our null findings stand in stark contrast to earlier meta-analyses reporting a significant association between the C242T polymorphism and ischemic cerebrovascular disease [21], 22]. This discrepancy is largely ascribed to a critical methodological refinement in our study. Previous analyses incorporated primary studies where control groups significantly deviated from Hardy-Weinberg Equilibrium (HWE). Such deviations are a well-established indicator of potential genotyping errors, population stratification, or selection bias, which can significantly distort true genetic effect estimates in meta-analyses [41]. By rigorously excluding studies with HWE violations in controls, our analysis provides a more accurate and reliable pooled estimate. This methodological rigor aligns our results with the well-designed, null-finding study by Gu et al. [20] and highlights the critical importance of quality control in genetic association syntheses. Therefore, the principal contribution of this updated meta-analysis lies not merely in its recency, but in its application of a more stringent methodological standard, suggesting earlier positive associations may have been biased.

Furthermore, the relationship between the C242T polymorphism and ROS production is complex. Studies have shown that the T242 allele is associated with reduced NADPH oxidase activity and lower ROS levels in vascular cells [42], 43]. Arca et al. demonstrated that the T242 allele predicts a lower risk of recurrent cardiovascular events and is linked to reduced systemic oxidative stress [44]. In CC individuals, augmented NADPH oxidase activity was unrelated to elevated p22phox expression levels, implying that the C242T polymorphism disrupts the binding capacity of p22phox to gp91phox, thereby modulating NADPH oxidase function [42]. However, other research has reported no significant effect of the C242T polymorphism on oxidative stress levels in healthy individuals at rest [45]. These conflicting findings highlight the need for further research, particularly in larger and more diverse populations, to elucidate the role of this polymorphism in ischemic stroke.

The striking reduction in heterogeneity observed within specific subgroups, such as small-vessel occlusion (SVO) and Caucasian populations, warrants further consideration. Biologically, this may indicate a more homogeneous pathogenic role for NADPH oxidase-derived ROS in these distinct stroke etiologies or genetic backgrounds. For instance, the pathophysiology of SVO closely links to cerebral microangiopathy and endothelial dysfunction, processes where NADPH oxidase activity is profoundly implicated. The absence of significant heterogeneity in Caucasians could reflect a more uniform genetic architecture and a lower degree of population stratification compared to more diverse ethnic groups, thereby simplifying the genetic association and thus reinforcing this specific link.

Several limitations of our meta-analysis should be acknowledged. First, our findings rest primarily on unadjusted effect estimates. A notable limitation of this approach is its failure to adjust for potential confounding variables like age, hypertension, smoking status, diabetes, and lipid profiles. These factors are established independent predictors of IS and may also influence oxidative stress levels, thereby acting as confounders or effect modifiers in the genotype-phenotype relationship. The primary studies’ lack of adjustment for these variables introduces the risk that residual confounding could bias our pooled estimates. Consequently, the null association we observed might mask a subtle genuine association obscured by confounding, or conversely, bolster the evidence for a true null finding if such pervasive confounding existed yet still yielded no signal. Future meta-analyses incorporating individual-level data or adjusted estimates would be critical for providing more precise and confounder-resistant summary estimates. Second, our analysis is confined to published studies, which may introduce publication bias. The inclusion of unpublished studies with nonsignificant or negative results could alter the pooled estimate and provide a more balanced understanding of the true association. Third, the generalizability of our conclusions may be limited due to the ethnic diversity of the included studies. Only two studies focused exclusively on Caucasian populations, restricting statistical power and applicability to other ethnic groups. Fourth, while our meta-analysis found no statistically significant correlation, it is possible that a small true effect exists below the detection threshold of the available sample size, or that complex gene-environment interactions obscured a true relationship. Finally, the significant statistical heterogeneity across studies may affect the precision and robustness of our pooled estimate.

In summary, our meta-analysis suggests that the NADPH oxidase p22phox C242T polymorphism does not confer a significant risk for ischemic stroke. However, further large-scale studies with diverse populations are needed to validate these findings. Additionally, exploring potential interactions between the p22phox C242T polymorphism and other genetic or environmental factors may provide deeper insights into its role in ischemic stroke susceptibility.

## Supplementary Material

Supplementary Material
