# Supplementary material for: Revisiting the link between NADPH oxidase p22phox C242T polymorphism and ischemic stroke risk: an updated meta-analysis
Source: Open Life Sci. 2025 Dec 30;20(1):20251221. doi: 10.1515/biol-2025-1221 (PMC13011899; doi:10.1515/biol-2025-1221)
Supplement: Supplementary file 1 — Supplementary Material [file j_biol-2025-1221_suppl_001.doc]

# **Revisiting the Link Between NADPH Oxidase p22phox C242T Polymorphism and Ischemic Stroke Risk: An Updated Meta-Analysis**

**Supplementary Material**

Table S1. Baseline characteristics of excluded studies

| First author,  Year of publication | Country | Ethnicity | Sample Size | | Age(Mean±SD,y) | | Reasons |
| --- | --- | --- | --- | --- | --- | --- | --- |
|  |  |  | Case | Control | Case | Control |  |
| Khan,2007 | UK | Caucasian | 274 | 406 | 67.81±10.22 | 67.13±8.21 | Insufficient data |
| Chen,2011 | China | East Asian | 176 | 131 | 65±9.4 | 62±9.1 | HWE deviation |
| Genius,2008 | Germany | Caucasian | 161 | 136 | 40.4±7.6 | 34.3±9.5 | HWE deviation |
| Sharma,2014 | India | Kashmiri | 43 | 120 | NA | NA | HWE deviation |
| Ji,2001 | China | East Asian | 81 | 74 | 61.7±1.0 | 58.4±1.2 | HWE deviation |

**References:**

[1]Khan U, Bevan S, Markus HS. NADPH oxidase polymorphisms in cerebral small vessel disease. Cerebrovasc Dis. 2007;24(1):135-8.

[2]Chen N, Fu Y, Ying YY, Ni PH. Correlation between NAD(P)H oxidase p22phox C242T polymorphism and cerebral infarction. Lab Med 2011; 26(03): 175–79.[Chinese]

[3]Genius J, Grau AJ, Lichy C. The C242T polymorphism of the NAD(P)H oxidase p22phox subunit is associated with an enhanced risk for cerebrovascular disease at a young age. Cerebrovasc Dis. 2008;26(4):430-3.

[4]Sharma A, Saleem S, Verma S, Afroze D. C242T Polymorphism of P22phox Gene of the Nox: A Putative Pathological Risk Factor for Stroke in Kashmiri Population. J Neurol Stroke 2014; 1(4):00020.

[5]Ji Z, Lu BX. Relationship of the C242T p22phox Gene Polymorphism to Cerebral Infarction; 2001 [Thesis].

Table S2. Subgroup analysis according to ethnicity.

| **Model** | **Ethnicity** | **Numberofstudies** |  | **Testofassociation** |  |  | **Testofheterogeneity** |  | **Publicationbias** |
| --- | --- | --- | --- | --- | --- | --- | --- | --- | --- |
|  |  |  | **OR** | **95%CI** | **p-val** | **Model** | **p-val** | **I^2** | **p-val**  **(Egger'stest)** |
| Allele contrast (T vs. C) | Overall | 10 | 1.2170 | [0.9613; 1.5408] | 0.102635125 | Random | 0.0004 | 0.7043 | 0.0352 |
|  | Caucasian | 2 | 1.0595 | [0.8990; 1.2486] | 0.490510259 | Fixed | 0.9298 | 0 | NA |
|  | East Asian | 8 | 1.3697 | [0.9713; 1.9315] | 0.072814057 | Random | 0.0001 | 0.7698 | 0.0173 |
| Recessive model (TT vs. TC+CC) | Overall | 8 | 1.1266 | [0.8404; 1.5104] | 0.425356042 | Fixed | 0.7962 | 0 | 0.5443 |
|  | Caucasian | 2 | 1.1532 | [0.8211; 1.6198] | 0.410731605 | Fixed | 0.7182 | 0 | NA |
|  | East Asian | 6 | 1.0525 | [0.5891; 1.8802] | 0.862860447 | Fixed | 0.6002 | 0 | 0.2454 |
| Dominant model (TT+TC vs. CC) | Overall | 10 | 1.2326 | [0.9537; 1.5931] | 0.110041336 | Random | 0.0008 | 0.6847 | 0.0188 |
|  | Caucasian | 2 | 1.0427 | [0.8397; 1.2947] | 0.705209504 | Fixed | 0.9139 | 0 | NA |
|  | East Asian | 8 | 1.3680 | [0.9607; 1.9482] | 0.082302322 | Random | 0.0002 | 0.7547 | 0.0165 |
| Overdominant (TC vs. TT + CC) | Overall | 10 | 1.1895 | [0.9313; 1.5193] | 0.164624707 | Random | 0.0029 | 0.6406 | 0.0121 |
|  | Caucasian | 2 | 0.9825 | [0.7882; 1.2246] | 0.874884655 | Fixed | 0.7277 | 0 | NA |
|  | East Asian | 8 | 1.3170 | [0.9424; 1.8405] | 0.106820286 | Random | 0.0009 | 0.7165 | 0.0163 |
| TT vs. CC | Overall | 8 | 1.1297 | [0.8340; 1.5302] | 0.430904258 | Fixed | 0.7509 | 0 | 0.4724 |
|  | Caucasian | 2 | 1.1590 | [0.8120; 1.6543] | 0.416258227 | Fixed | 0.7831 | 0 | NA |
|  | East Asian | 6 | 1.0550 | [0.5901; 1.8863] | 0.856606556 | Fixed | 0.5353 | 0 | 0.2293 |
| TT vs. TC | Overall | 8 | 1.1003 | [0.8073; 1.4996] | 0.5451259 | Fixed | 0.982 | 0 | 0.4797 |
|  | Caucasian | 2 | 1.1460 | [0.7980; 1.6459] | 0.460506716 | Fixed | 0.6676 | 0 | NA |
|  | East Asian | 6 | 0.9847 | [0.5418; 1.7899] | 0.959781218 | Fixed | 0.9502 | 0 | 0.8582 |
| TC vs. CC | Overall | 10 | 1.2024 | [0.9375; 1.5423] | 0.146655736 | Random | 0.0026 | 0.6448 | 0.014 |
|  | Caucasian | 2 | 1.0117 | [0.8032; 1.2743] | 0.921567066 | Fixed | 0.8002 | 0 | NA |
|  | East Asian | 8 | 1.3242 | [0.9442; 1.8571] | 0.103667469 | Random | 0.0007 | 0.7223 | 0.0162 |


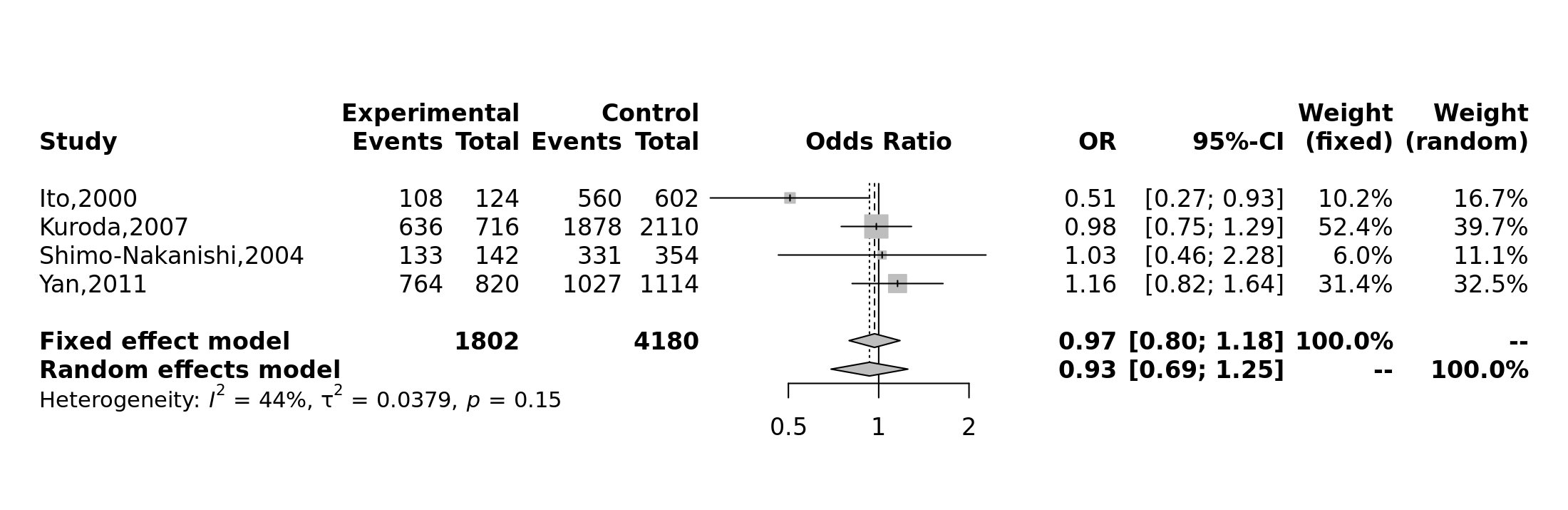


Figure S1. Forest plot analysis evaluating the association between NADPH Oxidase p22phox C242T polymorphism and large-artery atherosclerosis ischemic stroke risk (Allelic model).


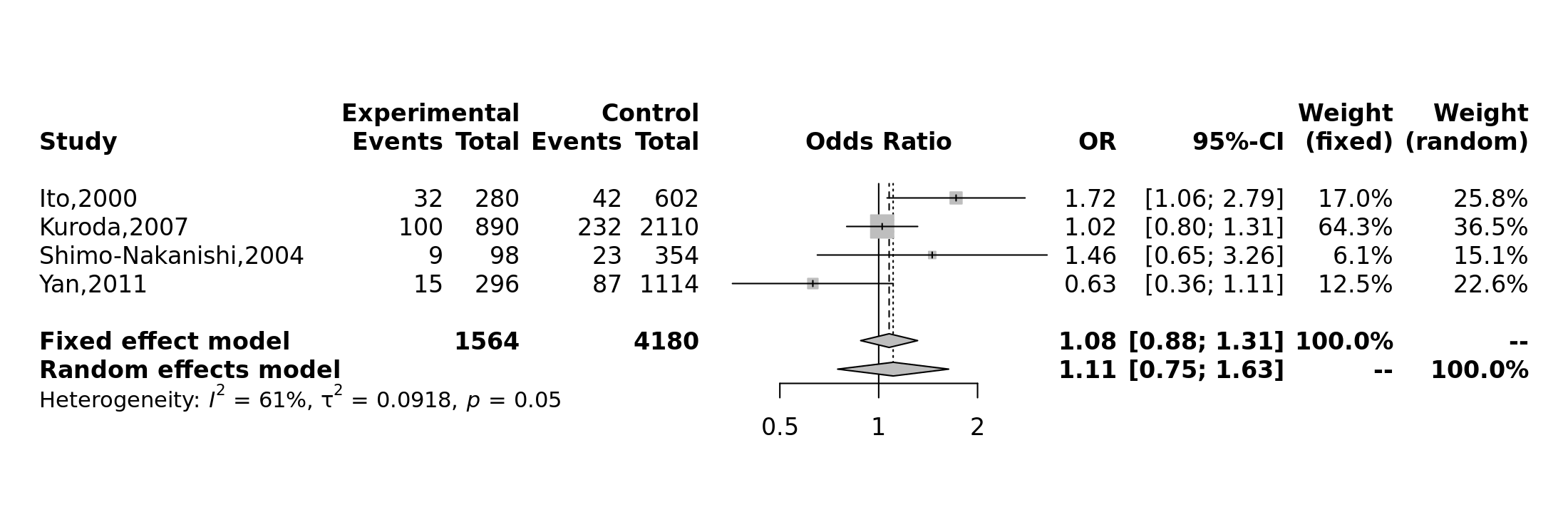


Figure S2. Forest plot analysis evaluating the association between NADPH Oxidase p22phox C242T polymorphism and small-vessel occlusion ischemic stroke (IS) risk (Allelic model).
